# Immune modulation of buffalo peripheral blood mononuclear cells by two asparaginyl endopeptidases from *Fasciola gigantica*

**DOI:** 10.1186/s13071-024-06570-5

**Published:** 2024-12-18

**Authors:** Dong-Qi Wu, Yan-Feng Guo, Yu Zou, Xiao-Ting Tang, Wei-Yu Zhang, Wen-Da Di

**Affiliations:** College of Animal Science and Technology, Guangxi University, Nanning, 530004 Guangxi Zhuang Autonomous Region People’s Republic of China

**Keywords:** *Fasciola gigantica*, Immunoregulation, Legumain, Peripheral blood mononuclear cell

## Abstract

**Background:**

Fascioliasis is a zoonotic parasitic disease caused by *Fasciola hepatica* and *Fasciola gigantica*, which poses a serious threat to global public health and livestock farming. *Fasciola gigantica* secretes and excretes various components to manipulate the immune response, thereby enhancing its invasion, migration, and survival in vivo. However, the roles of specific components in immune modulation, such as asparagine endopeptidase, remain unknown.

**Methods:**

The transcriptional abundance of members of the asparagine endopeptidase family (also known as the legumain family) from *F. gigantica* was analyzed. Two highly transcribed asparagine endopeptidases in metacercariae, juveniles and adults were cloned, and their recombinant proteins—recombinant *F. gigantica* legumain (r*Fg*LGMN-1) and (r*Fg*LGMN-2)—were expressed in prokaryotic expression system. Their regulatory effects on buffalo peripheral blood mononuclear cells (PBMCs), including proliferation, migration, total nitric oxide (NO) production, cytokine secretion, and phagocytosis were explored in vitro.

**Results:**

Ten members of the legumain family were detected in *F. gigantica*, among of which *Fg*LGMN-1 and *Fg*LGMN-2 exhibited high transcription levels in juveniles and adults. The isolation of sequences indicated that *Fg*LGMN-1 encodes 409 amino acids, while *Fg*LGMN-2 encodes 403 amino acids. Both recombinant *Fg*LGMN-1 (r*Fg*LGMN-1) and r*Fg*LGMN-2 were recognized by serum from buffaloes infected with *F. gigantica*. Both r*Fg*LGMN-1 and r*Fg*LGMN-2 inhibited the proliferation of PBMCs, and r*Fg*LGMN-1 also inhibited the migration of PBMCs. While r*Fg*LGMN-1 increased the production of total NO, r*Fg*LGMN-2 decreased NO production. Both r*Fg*LGMN-1 and r*Fg*LGMN-2 increased the transcription of the cytokines interleukin-10 and transforming growth factor β. The effect of r*Fg*LGMN-1 and r*Fg*LGMN-2 on the phagocytosis of PBMCs varied depending on their concentrations.

**Conclusions:**

r*Fg*LGMN-1 and r*Fg*LGMN-2 modulate several cellular and immunological functions of PBMCs, and exhibited distinct regulatory effects on these in vitro, which indicated that they may play roles in immune modulation and facilitate fluke development. However, due to uncertainties associated with in vitro experiments, further studies are necessary to elucidate the precise functions of these legumains.

**Graphical Abstract:**

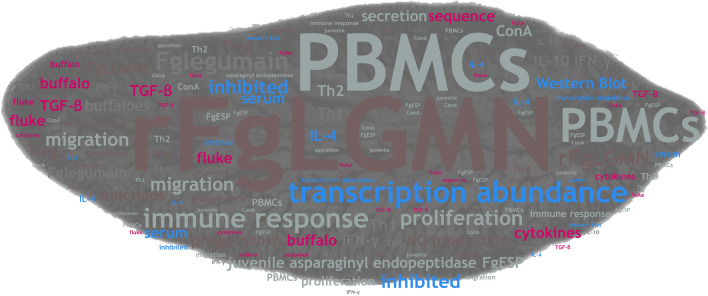

**Supplementary Information:**

The online version contains supplementary material available at 10.1186/s13071-024-06570-5.

## Background

Fascioliasis, a neglected tropical disease caused by *Fasciola hepatica* and *Fasciola gigantica*, results in significant economic losses in livestock production and poses substantial health risks to humans globally. Annual economic losses due to *Fasciola* infections are estimated at US $ 3 billion [1], with approximately 2.4 million people currently infected and 180 million at risk of infection [2]. Anthelmintic resistance in certain strains of *Fasciola* has emerged following prolonged use of triclabendazole and other drugs [3, 4]. Moreover, potential hybridization between *F. hepatica* and *F. gigantica* may lead to the production of more virulent offspring, thereby complicating disease management [5]. Concerns have also arisen regarding chemical residues in animal products resulting from treatment, which necessitates the exploration of novel immunotherapeutic strategies. During the migration and development of *F. gigantica* within a host, the host initiates immune responses that are aimed at resisting the fluke’s invasion [6, 7]. However, *F. gigantica* adeptly manipulates these responses to its advantage by suppressing inflammation and modulating macrophage polarization, thereby enhancing its survival [8]. These mechanisms primarily involve *F. gigantica* excretory-secretory products (*Fg*ESPs) [9], and highlight the intricate host-parasite interactions in *Fasciola* infections.

*Fg*ESPs encompass various components that have been systematically characterized, as members of the 14-3-3 protein family (specifically 14-3-3e), thioredoxin peroxiredoxin (TPX) and calcium-binding EF-hand protein 4 are recognized for their pivotal roles in modulating host immune responses to *F. gigantica* infection [10–12]. r*Fg*14-3-3e plays a critical role in the recognition of *F. gigantica* by innate immune cells, and modulates the cytokine profile of goat peripheral blood mononuclear cells (PBMCs). It promotes the secretion of interleukin-10 (IL-10) and transforming growth factor β (TGF-β), while suppressing the secretion of interleukin-4 (IL-4) and interferon-γ (IFN-γ), leading to an overall suppression of the immune response and a reduction in host inflammatory pathology [11]. Tian et al. [13] demonstrated that *Fg*TPX enhances the secretion of IL-2, IL-4, TGF-β, and IFN-γ, while inhibiting PBMCs proliferation and phagocytosis in vitro, thereby facilitating fluke infection. *Fg*Rab10 promotes PBMCs apoptosis and migration, enhances monocyte phagocytosis, and plays a multifaceted role in maintaining host immune homeostasis [14]. Asparaginyl endopeptidase, also known as legumain, exhibits specificity in hydrolyzing peptides and proteins at asparagine residues [15, 16]. Legumain has been identified in a wide range of vertebrates (including humans, cattle, pigs, *Haemaphysalis longicornis* and mice) and invertebrates (such as *F. gigantica*, *Schistosoma mansoni*, *Angiostrongylus cantonensis*, and *Haemonchus contortus*) [17–21]. The role of legumain in regulating the activation of cathepsin L and cathepsin B has been highlighted [22], as recombinant *S. mansoni* asparaginyl endopeptidase could transactivate the *S. mansoni* cathepsin B1 (CB1) pro-enzyme into its mature catalytic form in vitro. Zhang et al. [23] found that the transcription abundance of cathepsin L and cathepsin B were high during parasitism by *F. gigantica* and that asparagine endopeptidase showed a similar transcription pattern, suggesting the potential for legumains (LGMNs) to act as the rapeutic targets for diseases such as fascioliasis [24].

The role of legumains in *Fasciola* has been preliminarily explored, with Adisakwattana et al. [17] demonstrating that *Fg*LGMN-1 and *Fg*LGMN-2 are localized in the intestinal epithelium of the fluke and are transcribed during the developmental stages from newly excysted juvenile to adult stage. Robinson et al. [25] identified asparaginyl endopeptidases in the ESPs of newly excysted juveniles and 21-day-old juveniles of *F. hepatica*. However, the immunomodulatory effects of *Fg*LGMNs on the host remain unclear. 

In the present study, screening the whole genome sequences of *F. gigantica* revealed the presence of 10 legumain members. Among these, *FgLGMN-1* and *FgLGMN-2* exhibited high transcription levels in metacercariae, juveniles, and adults, suggesting their potential as candidates for drug and vaccine development. Subsequently, the immune regulatory functions of recombinant *Fg*LGMN-1 and *Fg*LGMN-2 on buffalo PBMCs were investigated in vitro. This study aims to deepen our understanding of the role of *Fg*LGMNs in immune regulation during *F. gigantica* infection and lays a foundation for the prevention of fascioliasis.

## Methods

### Ethics statement and buffalo maintenance

All experimental procedures involving the use of animals were approved by the Animal Ethics Committee of Guangxi University. The buffaloes were stall-fed a balanced diet at the dairy facility of the Buffalo Research Institute, Chinese Academy of Agricultural Sciences. *Fasciola gigantica*-infected buffalo sera and *F. gigantica*-negative buffalo sera were collected and kept in the lab. Briefly, whole blood samples were collected, and they were incubated at 37 °C for 1 h, and then the liquid was centrifuged at 3000 r.p.m. for 20 min at 4 °C for supernatant collection. Subsequently, the serum layer was collected and stored at − 80 °C until use.

### Confirmation of the absence of *Fasciola gigantica* infection in buffaloes

The absence of *F. gigantica* infection was confirmed by carrying out indirect ELISA on *Fg*ESP twice, with an interval of 4 weeks between the assays. *Fg*ESP was prepared as previously described [23], and indirect ELISA was performed as follows, with three replicates per sample. *Fg*ESP was diluted with 0.05 M carbonate buffer to 2.5 µg/mL, and 100 µL was added to the microplate wells, followed by incubation at 4 °C overnight. Then the microplate was washed three times with phosphate-buffered saline (PBS) with Tween 20 (PBST) (1 L of 0.01 M PBS solution added together with 500 µL Tween-20), and 1% gelatin (1 g gelatin was added to 100 mL of 0.01 M phosphate buffer) was added and the microplate incubated at 37 °C for 2 h. Subsequently, 100 µL buffaloes-serum that was diluted with phosphate at a dilution of 1:400 was added and the microplate incubated at 37 °C for 2 h. After washing the microplate with PBST, the diluted enzyme-labeled secondary antibody (1:20,000) (horseradish peroxidase-labeled goat secondary antibody IgG; (BIO-RAD, California) was added and the microplate incubated at 37 °C for 1 h. It was then washed with PBST, 100 µL TMB chromogenic solution (Solarbio, Beijing, China) was added, and incubated at 37 °C for 15 min, before 50 µL termination solution (2 M H_2_SO_4_ solution) was added. The optical density (OD) at a wavelength of 450 nm (OD_450nm_) value was measured and the P/N ratio calculated and compared with the critical values. The buffaloes that were negative for primary indirect ELISA were subjected to PBMCs collection. The buffaloes that were negative for both of the indirect ELISA were confirmed to be free of *F. gigantica* infection.

### Transcription abundance of *FgLGMNs* and predicted secretory *Fg*LGMNs

Transcriptomic data for different developmental stages of *F. gigantica* had been previously generated by RNA-seq. The transcription of the legumain genes was calculated by the fragments per kilobase per million reads method [26], which was used to compare the differences in gene transcription levels among different development stages. False Discovery Rate (FDR) control is a statistical method that was used to correct for p-value. Genes with an adjusted p-value<0.05 found by DESeq were assigned as differentially transcriped. The transcription data were analyzed, clustered, and visualized using the online program Cluster heatmap (https://cloud.oebiotech.com/task/detail/heatmap/). The 10 screened *LGMN* transcript sequences were also subjected to alignment with 15 previously characterized *LGMN* sequences in WormBase (accession numbers FGIG_05822, FGIG_03223, FGIG_03222, FGIG_01656/FGIG_01657, FGIG_02692, FGIG_02693, FGIG_02694, FGIG_02692, FGIG_05552, FGIG_01660, FGIG_12115, FGIG_10511, FGIG_05551, FGIG _ 01940, FGIG _10510/FGIG_10509).

For *Fg*LGMNs, the classic secretory protein was predicted by the programs SignalP 5.0, TargetP, and TMHMM [27–29], while the non-classic secretory protein was predicted by the SecretomeP—2.0 program [30].

### *Fg*LGMN-1 and *Fg*LGMN-2 coding sequence cloning and molecular modeling

Adult flukes of *F. gigantica* were collected from the gall bladders of naturally infected buffaloes slaughtered at local abattoirs in the Guangxi Zhuang Autonomous Region, People’s Republic of China. After washing in PBS (pH 7.4), the flukes were immediately used for RNA isolation. Species identification was performed by polymerase chain reaction (PCR), with only confirmed *F. gigantica* specimens subjected to subsequent RNA isolation. For species identification, the second internal transcribed spacer (ITS-2) region of ribosomal DNA (rDNA) was amplified and sequenced [31]. The reaction reagents were prepared in a final volume of 25 μL containing 12.5 μL PCR Master Mix polymerase (Vazyme, Jiangsu, China), 2 μL template genomic DNA, 0.5 μL forward primer and 0.5 μL reverse primer, and 10 μL deionized water. The PCR protocol was set as follows: 95 °C for 5 min, 32 cycles of 94 °C for 30 s, 59 °C for 30 s, and 72 °C for 30 s, and final extension at 72 °C for 7 min. An aliquot (5 μL) of each sample was electrophoresed and then sent for sequencing (Sangon Biotech, Shanghai, China). The ITS-2 primer sequences are listed (Additional file 1: Table S1).

RNA isolation was performed using Trizol (TransGen Biotech, Beijing, China), and RNA purity was assessed with a Nanodrop 2000C spectrophotometer (Thermo Scientific, Waltham, MA). Complementary DNA (cDNA) was synthesized using a cDNA synthesis kit (Novi Zan Biotechnology, Beijing, China), and the cDNA was used for *FgLGMN-1* and *FgLGMN*−2 sequence amplification. Two pairs of primers, designed based on the coding sequences of *FgLGMN-1* (GenBank accession number EF206821.1) and *FgLGMN-2* (EF206822.1), were used for PCR amplification. The primers used are listed (Additional file 1: Table S1).

The full-length sequences of *Fg*LGMN-1 and *Fg*LGMN-2 were submitted to the Swiss-Model website (https://swissmodel.expasy.org/) for tertiary structure prediction. The modeling output with the highest similarity value and a global model quality assessment value closest to 1 was selected. The Protein Data Bank (PDB) files of the query sequences were visualized using PyMOL software.

### Expression and Detection of recombinant *Fg*LGMN-1 and recombinant *Fg*LGMN-2

The cloned coding sequences of *FgLGMN-1* and *FgLGMN*−2 were ligated into the pET-28a (+) vector using a kit (TransGen Biotech), and the resultant constructs named pET-28a-*Fg*LGMN-1 and pET-28a-*Fg*LGMN-2. The ligated products were then transformed into *Escherichia coli* BL21(DE3) competent cells and verified by sequencing (Sangon Biotech, Shanghai, China). The transformants were cultured and induced with 0.8 mol/L isopropyl-β-D-thiogalactopyranoside (Solarbio) to produce recombinant proteins r*Fg*LGMN-1 and r*Fg*LGMN-2. These recombinant proteins were purified using HisPur^™^ Ni–NTA Spin Columns (CWBIO, Jiangsu, China) and subjected to sodium dodecyl sulfate–polyacrylamide gel electrophoresis (SDS-PAGE).

Endotoxins from r*Fg*LGMN-1 and r*Fg*LGMN-2 were removed using the ToxinEraserTM endotoxin removal kit (L00338) (Genscript, Jiangsu, China). Briefly, the pre-packed column was set up, followed by the addition of 5 mL of regeneration buffer and 6 mL of equilibrium buffer to equilibrate the column. The r*Fg*LGMN-1 and r*Fg*LGMN-2 proteins were then introduced separately into the column, followed by the addition of an equilibrium buffer. The eluents, containing endotoxin-free r*Fg*LGMN-1 and r*Fg*LGMN-2, were collected. The endotoxin level was assessed using the ToxinSensor Limulus test kit (L00350C) (Genscript, Jiangsu, China). The endotoxin-free protein solutions were filtered through a 0.22-μm filter and stored at −80 °C for future use.

The prepared r*Fg*LGMN-1 and r*Fg*LGMN-2 were loaded onto a 12% SDS-PAGE gel, transferred to Hybond-C extra nitrocellulose membranes (Amersham Biosciences, MI), and blocked with 5% bovine serum albumin in PBST at 37 °C for 1 h. Buffalo antisera (1:400 dilution with PBST), both positive and negative for *F. gigantica*, were used as primary antibodies. Horseradish peroxidase)-conjugated rabbit anti-bovine immunoglobulin G (Abmart, Shanghai, China) (1:5000 dilution with PBST) was used as the secondary antibody. The Western blot was developed using 3,3'-diaminobenzidine (Sigma, USA) as a chromogenic substrate.

### Sources of PBMCs

Jugular venous blood from three *F. gigantica*-negative female buffaloes aged 2–3 years were aseptically collected into EDTA-K2 vacuum collection tubes, mixed, and centrifuged at 2000 r.p.m. for 20 min. The white membrane layer was collected and centrifuged at 2000 r.p.m. for an additional 25 min after adding an equal volume of lymphocyte separation solution (HaoYang, Tianjin, China). The resulting white layer was collected and mixed with an equal volume of diluent and centrifuged at 1800 r.p.m. for 8 min. After discarding the supernatant, the cell precipitate was resuspended in cell diluent. The cells were then resuspended in 3 mL of RPMI-1640 complete medium, and the cell density was adjusted to 1 × 10^6^ cells/mL. Additionally, cell viability was assessed using the trypan blue exclusion test, and only cell populations with a viability > 95% were used for subsequent experiments.

### Effects of r*Fg*LGMN-1 and r*Fg*LGMN-2 on proliferation and migration of PBMCs

RPMI 1640 medium containing 1 × 10^6^ cells/mL was dispensed into each well of a 96-well tissue culture plate (100 µL/well). r*Fg*LGMN-1 and r*Fg*LGMN-2 at concentrations of 5, 10, 20, 40, and 60 µg/mL were added to the respective wells. Concanavalin A (ConA) (100 ng/mL) served as the positive control, while wells containing only cell culture medium served as the negative control. The plates were incubated in a 5% CO_2_ atmosphere at 37 °C for 48 h. Subsequently, 10 µL of CCK-8 reagent (Beyotime Biotechnology, Jiangsu, China) was added to each well, followed by a 4-h incubation. Absorbance was measured at OD_450_ using a microplate reader (Bio-Rad, Hercules, CA). The cell proliferation index was calculated as the ratio of treated cells OD_450_/control cells OD_450_.

For cell migration assays, cells were adjusted to a density of 1 × 10^6^ cells/mL and 1 mL of cell suspension was added to each well of a 24-well cell culture plate. Different concentrations (5, 10, 20, 40, and 60 μg/mL) of r*Fg*LGMN-1 and r*Fg*LGMN-2 were added to the respective wells. Plates were then incubated in a 5% CO_2_ incubator at 37 °C for 48 h. After incubation, cells were harvested and adjusted to a concentration of 1 × 10^5^ cells/mL. A total of 600 μL of complete medium was added to the lower chamber of Transwell migration chambers and 100 μL of the cell suspension was added to the upper chamber. The chambers were then incubated in a 5% CO_2_ incubator at 37 °C for 4 h. Cells that migrated to the lower chamber were collected and counted to determine the migration rate. All experiments were performed independently and repeated three times.

### Determination of total nitric oxide production and phagocytic activity

The production of total nitric oxide (NO) in the PBMC supernatant was determined using the Griess assay. PBMCs at a density of 1 × 10^6^ cells/mL were stimulated with various concentrations (5, 10, 20, 40, and 60 μg/mL) of r*Fg*LGMN-1 and r*Fg*LGMN-2 in RPMI-1640 medium and cultured in a 5% CO_2_ incubator at 37 °C for 24 h. Then culture supernatants were collected and subjected to the Griess reaction using the Griess detection kit (Beyotime Biotechnology, Beijing, China). The absorbance at OD_540_ was measured using a microplate reader (Bio-Rad) and converted to micromolar concentrations.

In a separate experiment, PBMCs were adjusted to a density of 1 × 10^6^ cells/mL and plated in 6-well plates. Cells were treated with different concentrations of r*Fg*LGMN-1 and r*Fg*LGMN-2 (5, 10, 20, 40, and 60 μg/mL) and cultured under 5% CO_2_ at 37 °C for 24 h. Following treatment, cells were washed with PBS, digested with trypsin (Solarbio, Shanghai, China) for 2 min, and re-suspended in RPMI-1640 complete medium (Gibco, Grand Island, NY). Phagocytosis assays were conducted using the FITC–Dextran phagocytosis kit (Becton Dickinson, NY) according to the manufacturer’s instructions. Briefly, cells were incubated with 100 μL of fluorescein isothiocyanate-dextran for 1 h, washed twice with PBS, and collected for the analysis of monocyte phagocytic activity by flow cytometry. All experiments were performed independently and repeated three times.

### Effects of r*Fg*LGMN-1 and r*Fg*LGMN-2 on cytokine transcription

The density of PBMCs was adjusted to 1 × 10^6^ cells/mL and the cells cultured in 6-well plates. The cells were treated with r*Fg*LGMN-1 and r*Fg*LGMN-2 (5, 10, 20, 40 and 60 μg/mL) for 24 h, while PBS was set as the control. The cells were washed with PBS and digested with trypsin solution (Solarbio). A RNA extraction kit (Solarbio) was then used for RNA extraction; 1 μg RNA was reverse transcribed according to the instructions for the PrimeScript RT reagent kit (Solarbio). The transcription abundances of IL-4, IL-10, IFN-γ, and TGF-β were determined by real-time qPCR; glyceraldehyde-3-phosphate dehydrogenase was used as the housekeeping gene. Real-time qPCR was performed according to the Fast Start Universal SYBR Green Master (Rox) (Roche, Basel, Switzerland) instructions. The reaction procedure was as follows: 95 °C for 30 s, followed by 40 cycles of 95 °C for 15 s, 56 °C for 15 s, and 72 °C for 30 s. Finally, the following conditions–95 °C for 15 s, 60 °C for 1 min and 95 °C for 15 s–were employed to generate the dissociation curve. The raw cycle thresholds (Cts) were obtained from the LightCycler application and then relative messenger RNA transcription abundance was calculated using the comparative Ct method with the formula 2−^ΔΔCt^ [32]. The primers used in the real-time qPCR are listed in Additional file 1: Table S1.

### Data analysis

Statistical analyses were conducted using GraphPad Prism software. One-way and two-way ANOVA were employed to assess statistical differences across various experimental conditions, with Tukey's test used for post hoc multiple comparisons. *P* < 0.05 was considered statistically significant. Data are presented as mean ± SD from three independent experiments (*n* = 3).

## Results

### Legumain transcript abundance and prediction of secretory *Fg*LGMNs

**Fig. 1:**
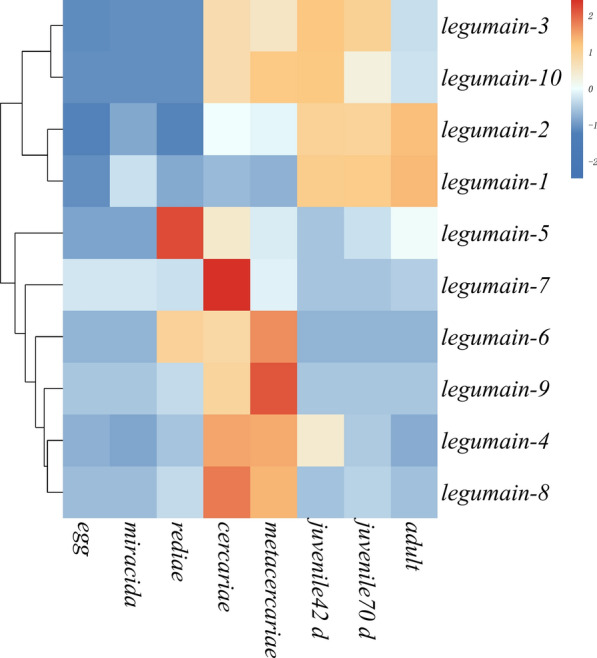
Transcription profile of legumain (*LGMN*) transcripts over the life cycle of *Fasciola gigantica*. Graphical representation of the 10 transcripts based on fragments per kilobase per million reads is shown as a heatmap. *Red* upregulation,* blue* downregulation

The alignment of 10 *LGMN* transcripts with 15 previously characterized LGMN sequences in WormBase indicated that 15 *LGMN* sequences constitutes 10 legumain sequences with complete sequences. Among the 10 LGMN transcripts, group A (*FgLGMN-3*, *FgLGMN*-*10*) exhibited high transcription levels in 42-day-old juveniles. Group B (*FgLGMN-1*, *FgLGMN-2*) showed high transcription in metacercariae, 42-day-old juveniles, 70-day-old juveniles, and adults. Group C (*FgLGMN-5*) was highly transcribed in rediae, while Group D (*FgLGMN-7*) was highly transcribed in cercariae. Group E (*FgLGMN-4*, *FgLGMN-6*, *FgLGMN-8*, *FgLGMN-9*) displayed had higher levels of transcription during the cercariae and metacercariae stages, compared to other stages of fluke development (low transcription levels across various stages of fluke development). (Fig. 1) (Additional file 2: Table S2). The 10 transcripts of *FgLGMNs* showed significant differential transcription across eight developmental stages (Additional file 3: Table S3).

All *Fg*LGMNs were predicted to be secretory proteins. *Fg*LGMN-1, *Fg*LGMN-2, *Fg*LGMN-5, *Fg*LGMN-8, *Fg*LGMN-9 and *Fg*LGMN-10 were predicted to be classic secretory proteins, whereas *Fg*LGMN-3, *Fg*LGMN-4, *Fg*LGMN-6 and *Fg*LGMN-7 were predicted to be non-classic secretory proteins.

### Molecular characterization of *Fg*LGMN-1 and *Fg*LGMN-2

The coding sequences of *FgLGMN-1* and *FgLGMN-2* were amplified and sequenced. *FgLGMN-1* showed 100% identity with the database sequence with GenBank accession number EF206821.1, while *FgLGMN-2* showed 98.96% identity with the database sequence with GenBank accession number EF206822.1. The coding sequence of *Fg*LGMN-1 comprises 1230 base pairs (bp), and encodes 409 amino acids, while the coding sequence of *Fg*LGMN-2 comprises 1212 bp, and encodes 403 amino acids.

**Fig. 2:**
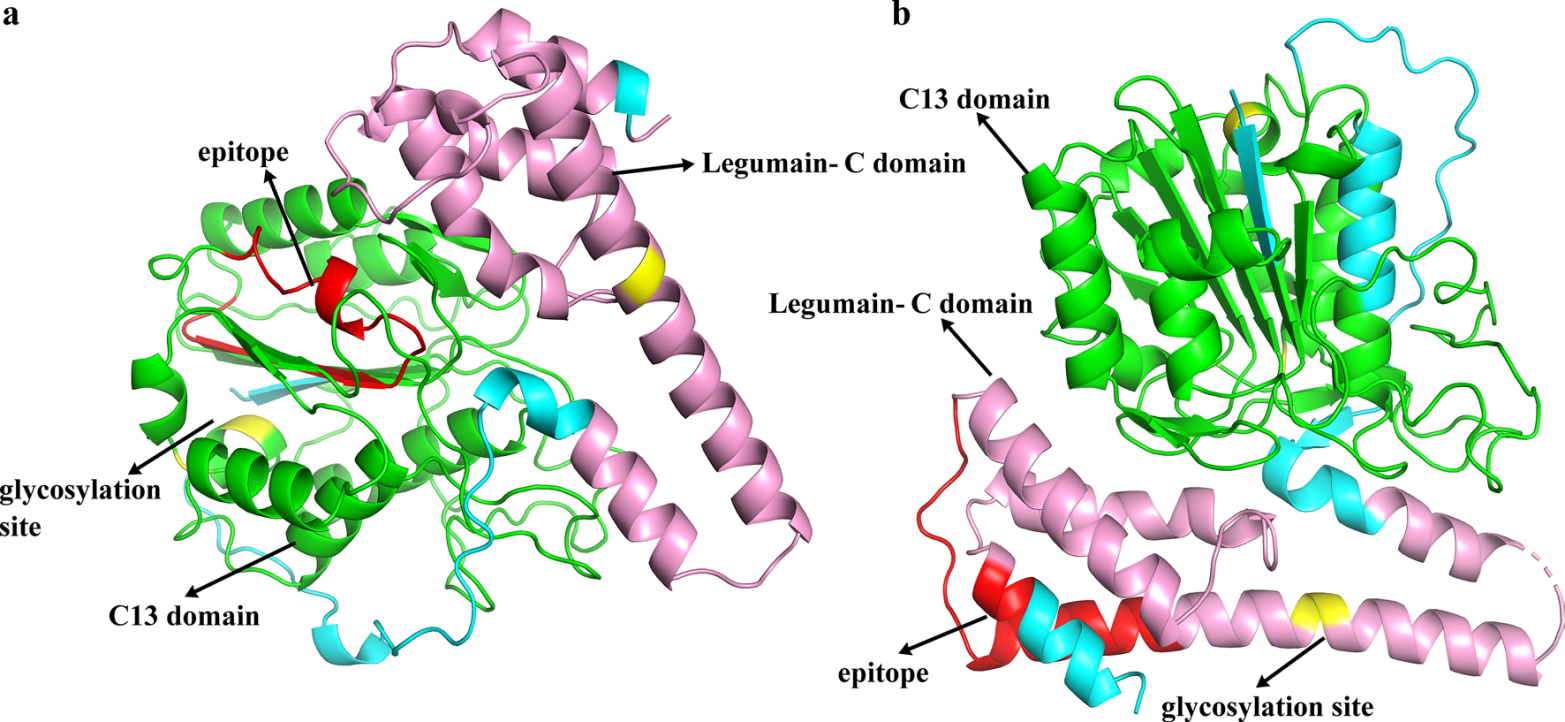
Putative tertiary structures of **a ***Fasciola gigantica* (*Fg*) LGMN-1 (*Fg*LGMN-1) and **b** *Fg*LGMN-2. Elements are color-coded, with peptidase C13 domain in* green*, LGMN-C domain in* pink*, the glycosylation sites in* yellow*, and the predicted epitope in* red*

The three-dimensional structure of human asparaginyl endopeptidase protein (PDB code Q99538) was used as a template to construct homology models of *Fg*LGMN-1 and *Fg*LGMN-2. As shown in Fig. 2, glycosylation sites were predicted at positions 261, 264, 279, and 341 of *Fg*LGMN-1, and at positions 277, 281, 341, and 325 of *Fg*LGMN-2. Antigenic epitopes were identified at positions 175–194 of *Fg*LGMN-1 and 348–367 of *Fg*LGMN-2. The C13 domain and LGMN-C domain are also marked.

### Expression and detection of r*Fg*LGMN-1 and r*Fg*LGMN-2

The *FgLGMN-1* and *FgLGMN-2* fragments (1227 bp for *FgLGMN-1* and 1209 bp for *FgLGMN-*2) were cloned into the pET-28a vector. The predicted molecular masses of r*Fg*LGMN-1 and r*Fg*LGMN-2 were 46 kDa and 43 kDa, respectively. Both r*Fg*LGMN-1 and r*Fg*LGMN-2 were expressed as His-tagged fusion proteins and detected by 12% SDS-PAGE after purification; they had molecular weights of approximately 49 kDa and approximately 46 kDa, respectively (Additional file 4: Figure S1).

Western blot analysis revealed that r*Fg*LGMN-1 migrated at approximately 49 kDa and was recognized by anti-*F. gigantica* serum from infected buffalo, but did not react with serum from uninfected buffalo. r*Fg*LGMN-2 migrated at approximately 46 kDa and was recognized by anti-*F. gigantica* serum from infected buffalo, but did not react with serum from uninfected buffalo (Additional file 4: Fig. S1).

### r*Fg*LGMN-1 and r*Fg*LGMN-2 inhibited the proliferation and migration of PBMCs

**Fig. 3:**
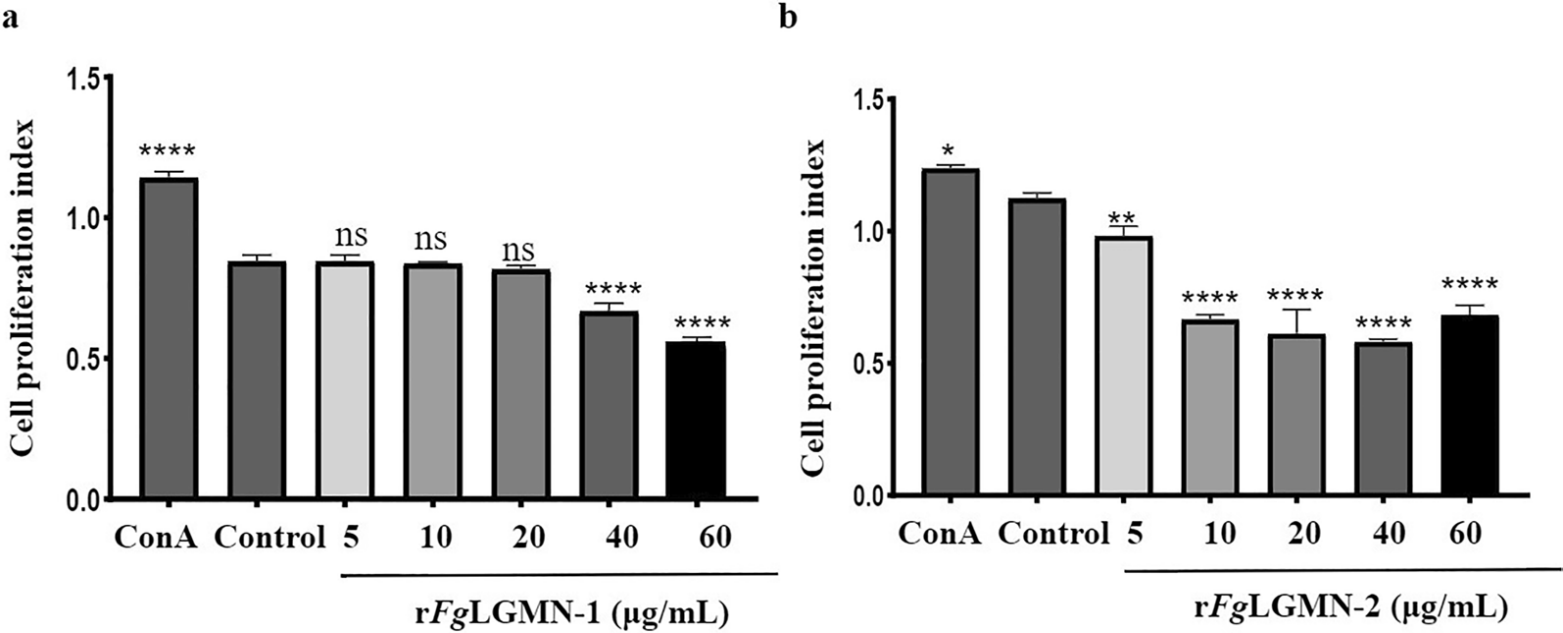
Effect of **a** r*Fg*LGMN-1 and **b** r*Fg*LGMN-2 on the proliferation of peripheral blood mononuclear cells (PBMCs). PBMCs treated with concanavalin A (ConA) and phosphate-buffered saline (PBS) were used as a control, and were also treated with different concentrations of r*Fg*LGMN-1 and r*Fg*LGMN-2 for 48 h. r*Fg*LGMN-1 and r*Fg*LGMN-2 inhibited the proliferation of PBMCs. Graphs represent means ± SDs of results from three independent biological replicates. Asterisks indicate statistically significant differences between treated cells and control cells: *P < 0.05, **P < 0.01, ****P < 0.0001,* ns* non-significant. For other abbreviations, see Figs. 1 and 2

For r*Fg*LGMN-1, as a positive control, ConA promoted cell proliferation [ANOVA, *F*_(6, 14)_ = 243.7, *P* < 0.001]. r*Fg*LGMN-1 at concentrations of 40 and 60 μg/mL significantly inhibited the proliferation of PBMCs (*P* < 0.0001). For r*Fg*LGMN-2, ConA promoted cell proliferation [ANOVA, *F*_(6, 14)_ = 134.3, *P* = 0.0178]. All tested concentrations of r*Fg*LGMN-2 significantly inhibited the proliferation of PBMCs (5 μg/mL vs control, *P* = 0.0032; 10, 20, 40 and 60 μg/mL vs control, *P* < 0.0001) (Fig. 3).

**Fig. 4:**
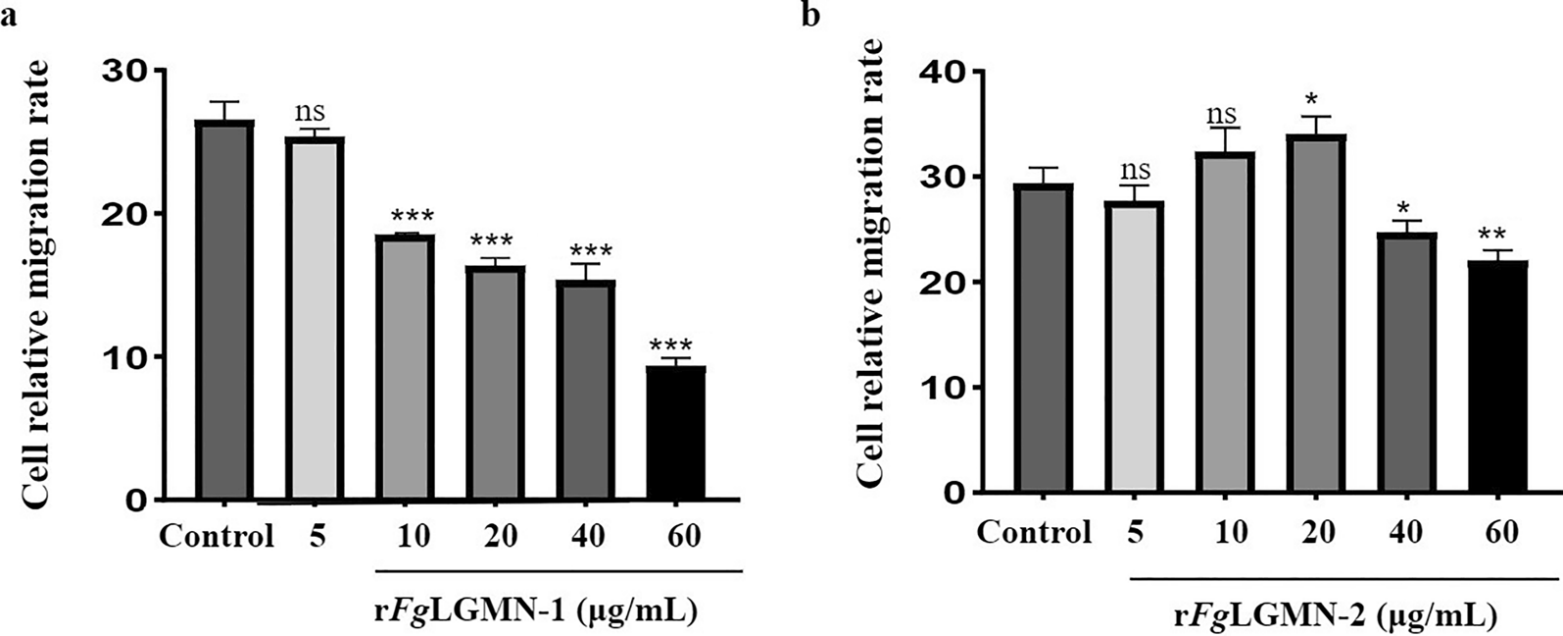
Effect of **a** r*Fg*LGMN-1 and **b** r*Fg*LGMN-2 on PBMC migration. PBMCs treated with PBS was performed as a control, and they were also treated with different concentrations of r*Fg*LGMN-1 and r*Fg*LGMN-2 for 24 h, respectively. Graphs represent means ± SDs of results from three independent biological replicates. Asterisks indicate statistically significant differences between treated cells and control cells: *P < 0.05, **P < 0.01, ***P < 0.001. For other abbreviations, see Figs. 1, 2, and 3

As shown in Fig. 4, r*Fg*LGMN-1 at concentrations of 10, 20, 40, and 60 μg/mL significantly inhibited PBMC migration [ANOVA, *F*_(5, 12)_ = 174.0, 10 μg/mL vs control, *P* = 0.0002; 20 μg/mL vs control, *P* = 0.0005; 40 μg/mL vs control, *P* = 0.0004; 60 μg/mL vs control, *P* = 0.0002]. r*Fg*LGMN-2 exhibited varying effects depending on its concentration; 20 μg/mL promoted cell migration [ANOVA, *F*_(5, 12)_ = 24.21, 20 μg/mL vs control, *P* = 0.0152], while 40 and 60 μg/mL inhibited cell migration (40 μg/mL vs control, *P* = 0.0152; 60 μg/mL vs control, *P* = 0.0005).

### Effects of r*Fg*LGMN-1 and r*Fg*LGMN-2 on NO production

**Fig. 5:**
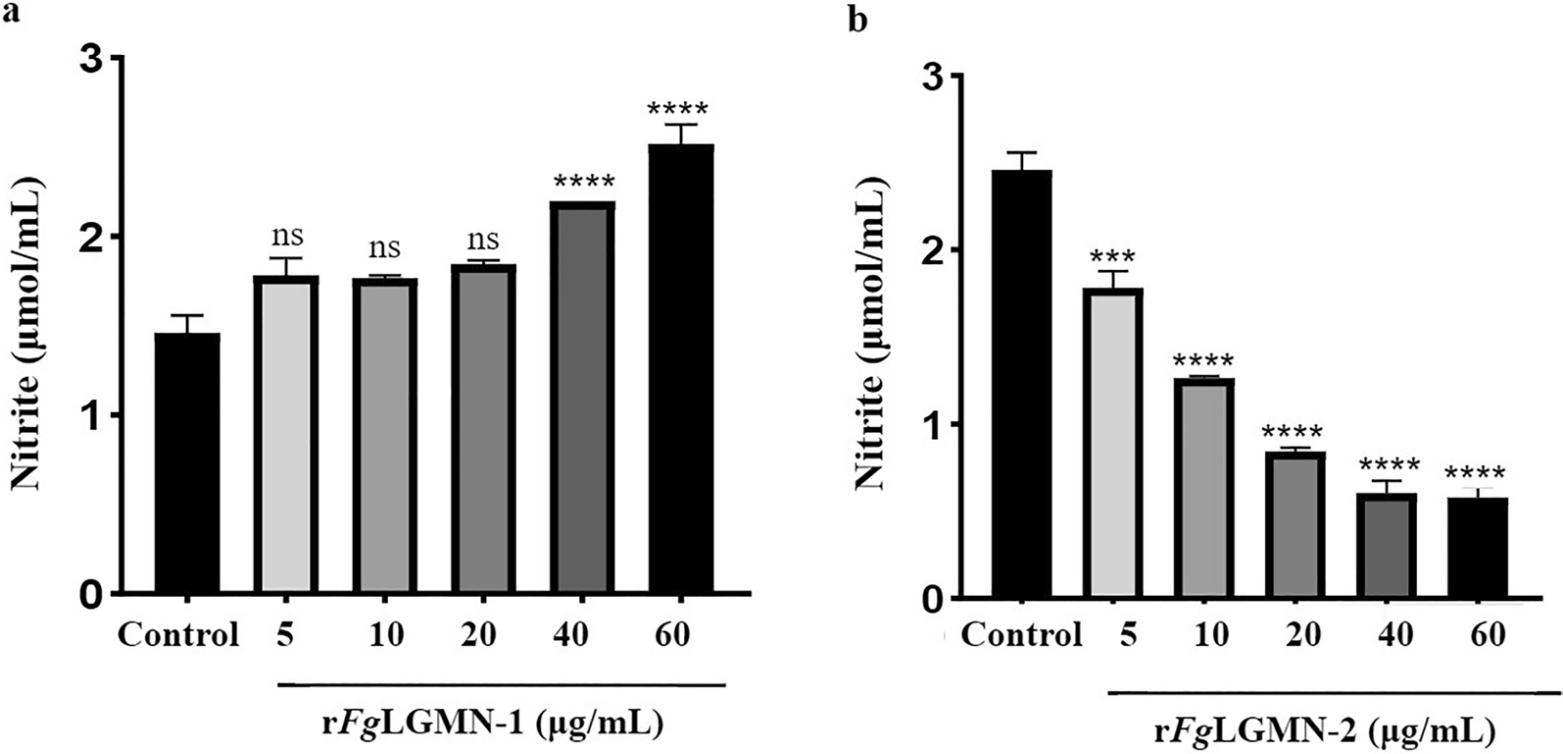
Effect of **a** r*Fg*LGMN-1 and **b** r*Fg*LGMN-2 on nitric oxide (NO) production. PBMCs treated with PBS was used as the control, and were also treated with different concentrations of r*Fg*LGMN-1 and r*Fg*LGMN-2 for 24 h, after which the cell supernatant was collected and NO was detected. Graphs represent means ± SDs of results from three independent biological replicates. Asterisks indicate statistically significant differences between treated cells and control cells: ***P < 0.001, ****P < 0.0001. For other abbreviations, see Figs. 1, 2, 3, and 4

For r*Fg*LGMN-1, 40 and 60 μg/mL promoted the production of NO [ANOVA, *F*_(5, 12)_ = 77.94, 40 and 60 μg/mL vs control, *P* < 0.0001]. For r*Fg*LGMN-2, all concentrations significantly inhibited NO production [ANOVA, *F*_(5, 12)_ = 29.84, 5 μg/mL vs control, *P* = 0.0037; 10, 20, 40 and 60 μg/mL vs control, *P* < 0.0001] (Fig. 5).

### Impacts of r*Fg*LGMN-1 and r*Fg*LGMN-2 on cytokine transcription

**Fig. 6:**
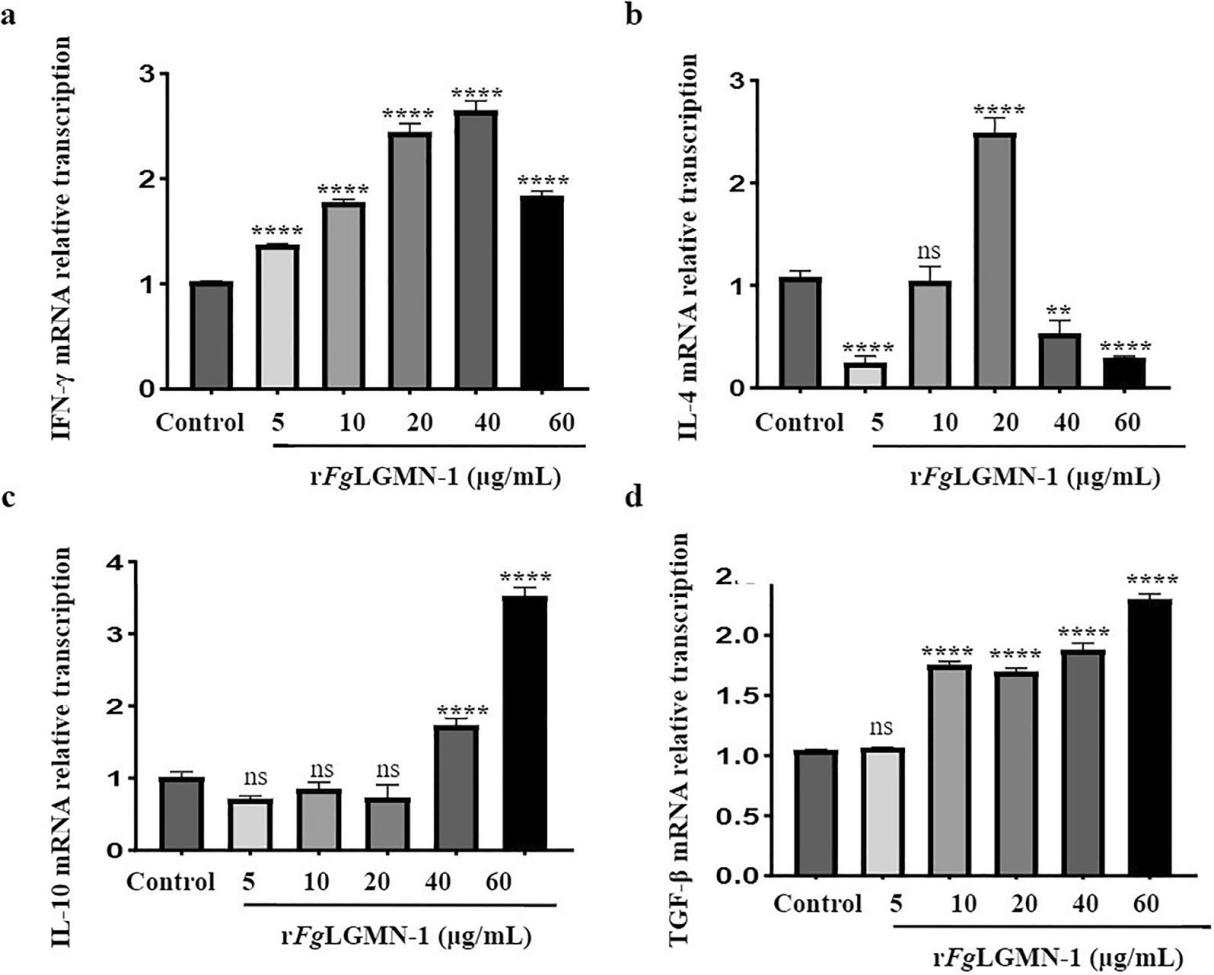
Effect of r*Fg*LGMN-1 on cytokine transcription. The messenger RNA abundance of cytokines, including **a** interferon-γ (IFN-γ), **b** interleukin-4 (IL-4), **c** IL-10 and **d** transforming growth factor β (TGF-β) in PBMCs stimulated by r*Fg*LGMN-1 were analyzed by real-time polymerase chain reaction (PCR); PBS-treated PBMCs served as the control. Graphs represent means ± SDs of results from three independent biological replicates. Asterisks indicate statistically significant differences between treated cells and control cells: **P < 0.01, ****P < 0.0001. For other abbreviations, see Figs. 1, 2, 3, 4, and 5

**Fig. 7:**
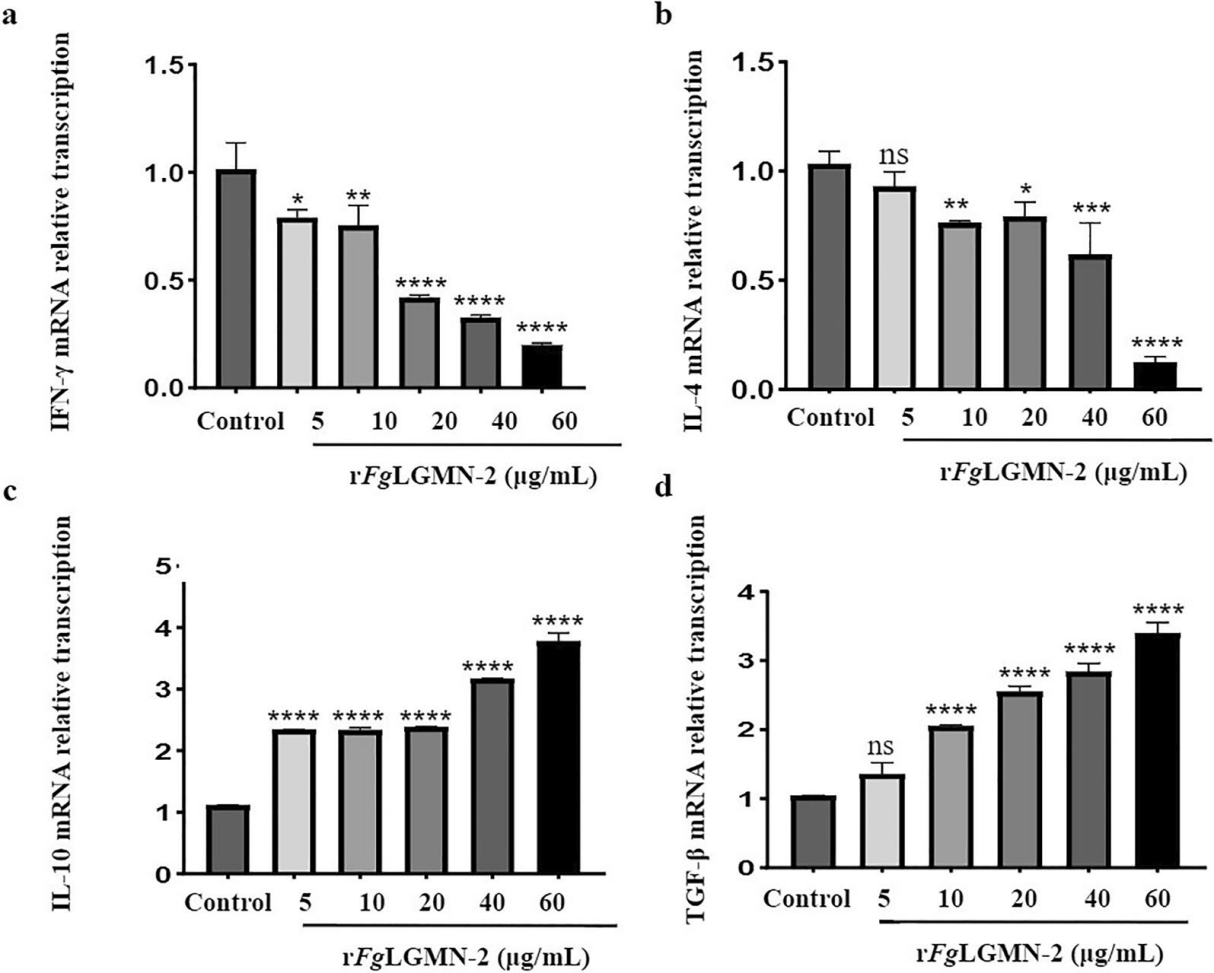
Effect of r*Fg*LGMN-2 on cytokine transcription. The messenger RNA abundance of cytokines, including **a** IFN-γ, **b** IL-4, **c** IL-10 and **d** TGF-β in PBMCs stimulated by r*Fg*LGMN-2, were analyzed by real-time PCR; PBS-treated PBMCs served as the control. Graphs represent means ± SDs of results from three independent biological replicates. Asterisks indicate statistically significant differences between treated cells and control cells: *P < 0.05, **P < 0.01, ***P < 0.001, ****P < 0.0001. For abbreviations, see Figs. 1, 2, 3, 4, and 5

The potential modulatory effects of r*Fg*LGMN-1 and r*Fg*LGMN-2 on PBMCs were investigated. As shown in Fig. 6, r*Fg*LGMN-1 promoted the transcription of IFN-γ, IL-10, and TGF-β. The transcription of IFN-γ was significantly upregulated with r*Fg*LGMN-1 treatment at 5, 10, 20, 40, and 60 μg/mL [ANOVA, *F*_(5, 12)_ = 356.2, *P* < 0.0001]. IL-10 transcription was significantly upregulated at 40 and 60 μg/mL of r*Fg*LGMN-1 [ANOVA, *F*_(5, 12)_ = 282.8, 40 and 60 μg/mL vs control, *P* < 0.0001]. TGF-β transcription was significantly upregulated at 10, 20, 40, and 60 μg/mL of r*Fg*LGMN-1 [ANOVA, *F*_(5, 12)_ = 588.6, 10, 20, 40 and 60 μg/mL vs control, *P* < 0.0001]. However, r*Fg*LGMN-1 displayed the opposite effect on IL-4 transcription depending on its concentration. A concentration of 20 μg/mL promoted the transcription of IL-4, and concentrations of 5, 40 and 60 μg/mL inhibited its transcription [ANOVA, *F*_(5, 12)_ = 189.0, 5, 40 and 60 μg/mL vs control, *P* < 0.0001; 40 μg/mL vs control, *P* = 0.0004] (Fig. 6).

r*Fg*LGMN-2 promoted the transcription of IL-10 and TGF-β, and inhibited the transcription of IFN-γ and IL-4. The transcription of IL-10 and TGF-β was significantly upregulated by all concentrations of r*Fg*LGMN-2 [for IL-10, ANOVA, *F*_(5, 12)_ = 716.0, *P* < 0.0001; for TGF-β, *F*_(5, 12)_ = 188.8, 10, 20, 40 and 60 μg/mL vs control, *P* < 0.0001]. All concentrations of r*Fg*LGMN-2 significantly inhibited the transcription of IFN-γ [*F*_(5, 12)_ = 70.45, 5 μg/mL vs control, *P* = 0.0114; 10 μg/mL vs control, *P* = 0.0037; 20, 40 and 60 μg/mL vs control, *P* < 0.0001]. Additionally, r*Fg*LGMN-2 at concentrations of 10, 20, 40, and 60 μg/mL significantly inhibited the transcription of IL-4 [*F*_(5, 12)_ = 53.89, 10 μg/mL vs control, *P* = 0.0089; 20 μg/mL vs control, *P* = 0.0196; 40 μg/mL vs control, *P* = 0.0002; 60 μg/mL vs control, *P* < 0.0001] (Fig. 7).

### Effect of r*Fg*LGMN-1 and r*Fg*LGMN-2 on phagocytosis

**Fig. 8:**
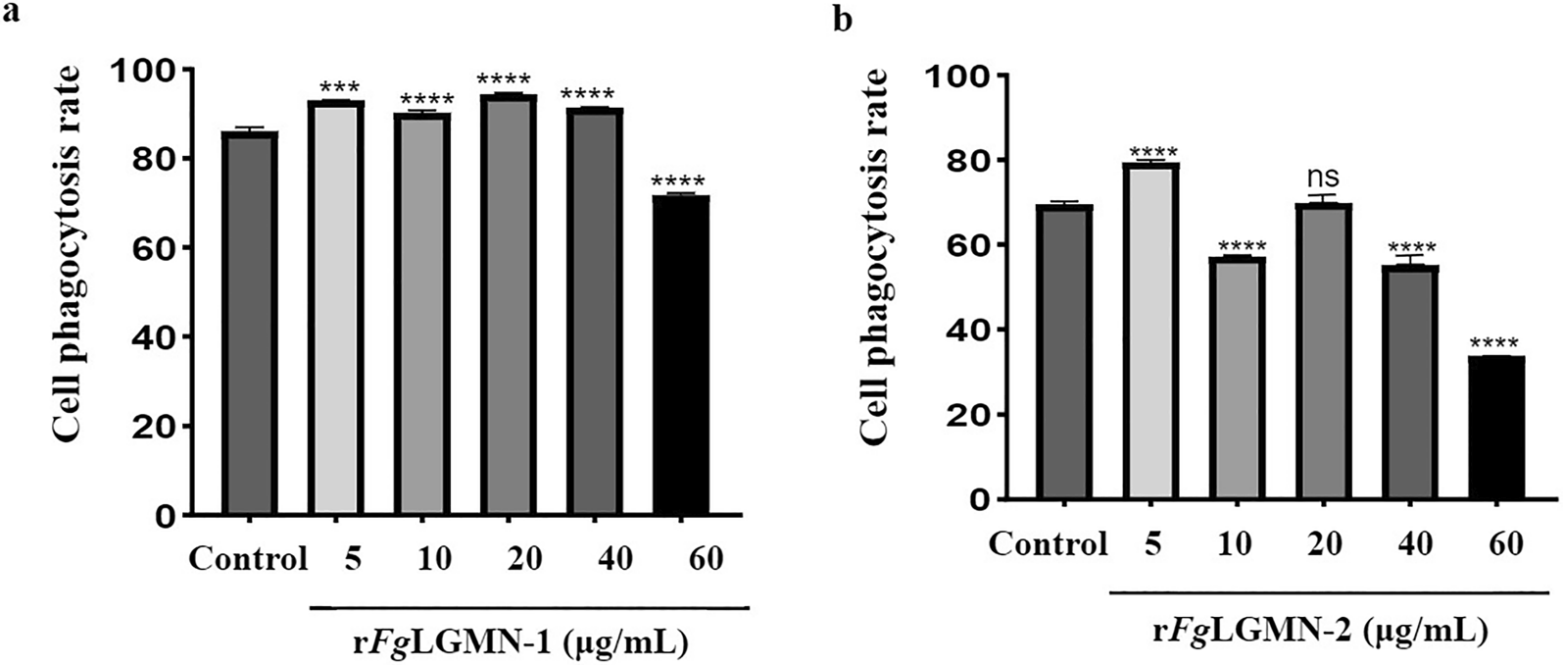
Effect of **a** r*Fg*LGMN-1 and **b** r*Fg*LGMN-2 on phagocytosis. PBMCs treated with PBS was used as the control, and were also treated with different concentrations of r*Fg*LGMN-1 and r*Fg*LGMN-2 for 24 h, after which the cell supernatant was collected and phagocytosis was determined. Graphs represent means ± SDs of results from three independent biological replicates. Asterisks indicate statistically significant differences between treated cells and control cells: ***P < 0.001, ****P < 0.0001. For abbreviations, see Figs. 1, 2, 3, 4, and 5

Both r*Fg*LGMN-1 and r*Fg*LGMN-2 displayed opposite effects on phagocytosis depending on their concentrations. For r*Fg*LGMN-1, 5, 10, 20 and 40 μg/mL promoted phagocytosis, while 60 μg/mL inhibited phagocytosis [*F*_(5, 12)_ = 664.7, 5 μg/mL vs control, *P* = 0.0004; 10, 20, 40 and 60 μg/mL vs control, *P* < 0.0001]. For r*Fg*LGMN-2, 5 μg/mL promoted phagocytosis, whereas higher concentrations (10, 40 and 60 μg/mL) inhibited phagocytosis [*F*_(5, 12)_ = 3693, 5 μg/mL vs control, *P* = 0.0037; 10, 40 and 60 μg/mL vs control, *P* < 0.0001] (Fig. 8).

## Discussion

Legumains are widely found in various types of parasites and are implicated in numerous biological and pathogenic processes [33–35]. They facilitate immune evasion through several mechanisms such as cleaving host immunoglobulins, inhibiting antigen presentation, and modulating the secretion of host cytokines and chemokines [36–38]. In the present study, screening of 10 *Fg*LGMNs from *F. gigantica* showed that they had diverse transcription profiles and all of them were predicted to be secretory proteins. Among these legumains, the transcription levels of *Fg*LGMN-1 and *Fg*LGMN-2 coding genes were notably high during the parasitic stage. Western blot analysis further confirmed that serum from *F. gigantica*-infected buffalo recognized both r*Fg*LGMN-1 and r*Fg*LGMN-2, which suggested their involvement in *F. gigantica*-buffalo interactions. Thus, the functions of *Fg*LGMN-1 and *Fg*LGMN-2 in the regulation of buffalo PBMCs including proliferation, migration, NO production, cytokine transcription, and phagocytic activity were explored.

### Transcription and sequence characterization of *Fg*LGMN-1 and *Fg*LGMN-2

Adisakwattana et al. [17] elucidated that *Fg*LGMN-1 and *Fg*LGMN-2 are synthesized in *F. gigantica* during its development from the newly excysted juvenile to the adult stage. In the present study, we found that both *FgLGMN*−1 and *FgLGMN* −2 showed a high level of transcription in the metacercariae and 42-day-old larvae, which is consistent with previous studies.

*Fg*LGMN-1 and *Fg*LGMN-2 displayed conserved LGMN-C and C13 domains, which are features of asparagine endopeptidase. In addition, the evaluation of the antigenic epitopes and glycosylation sites of the peptide structure indicated that *Fg*LGMN-1 and *Fg*LGMN-2 may undergo N-glycosylation modification. As the amino acids histidine, glycine, and cysteine were shown by Dall and Brandstetter [39] to be essential for enzyme catalysis, their presence in *Fg*LGMN-1 and *Fg*LGMN-2 may indicate that they also function in enzyme catalysis in these* Fasciola*. Specifically, cysteine and histidine are crucial for protease activity [40], and the glycine residue plays roles in the ligase activity of LGMN, indirectly facilitating substrate protonation by catalyzing histidine. Thus, cysteine 189 and histidine 148 may be crucial for *Fg*LGMN-1 and *Fg*LGMN-2 protease activity, and glycine 142 may play a role in ligase activity [41].

### *Fg*LGMN-1 and *Fg*LGMN-2 interfere with proliferation and migration of PBMCs

Fang Ni et al. [42] demonstrated that recombinant asparaginyl endopeptidase of *A. cantonensis* could be recognized by immunoglobulin G in the serum of mice and humans infected with *A. cantonensis*. Similarly, Robinson et al. [25] identified asparaginyl endopeptidases in the secretory proteins of immature migrating larvae (21 days old) in the liver. In the present study, bioinformatics analysis revealed that *Fg*LGMN-1 and *Fg*LGMN-2 are secretory proteins. This is consistent with their recognition by serum from *F. gigantica*-infected buffaloes, which further suggests that they potentially play roles in immune modulation. Although *Fg*LGMN-1 and *Fg*LGMN-2 exhibit similar functions in the promotion of proliferation and the transcription of regulatory T cell (Treg) cytokines (IL-10 and TGF-β), they show divergent effects on NO production and the transcription of the type 1 T helper (Th1) cytokine (IFN-γ).

Cell proliferation and migration are essential processes of the host's immune response [43]. Upon pathogen invasion, effector cell proliferation is stimulated in the host, facilitating the migration of these cells to the site of infection. ESPs from *F. hepatica* have been demonstrated to inhibit lymphoid cell proliferation in sheep and suppress the proliferation of spleen mononuclear cells in rats. In the present study, r*Fg*LGMN-1 and r*Fg*LGMN-2 similarly inhibited both the proliferation (40 and 60 μg/mL for r*Fg*LGMN-1, all concentrations for r*Fg*LGMN-2) and migration (10, 20, 40, 60 μg/mL for r*Fg*LGMN-1, 40 and 60 μg/mL for r*Fg*LGMN-2) of PBMCs. Comparable inhibitory effects have also been observed in other proteins, including *Fg*14-3-3e, *Fg*TPx, and *Fg*EF-hand protein 4 [11–13]. The reduced proliferation and migration of PBMCs in response to r*Fg*LGMN-1 and r*Fg*LGMN-2 may constitute an immunomodulatory strategy employed by *F. gigantica* to evade host immune defenses.

### *Fg*LGMN-1 and *Fg*LGMN-2 interfere with NO production and phagocytic activity of PBMCs

During fluke infection, the host activates various defense mechanisms, including the activation and polarization of macrophages [44], which exert killing effects through the inducible nitric oxide synthase-NO system [45]. NO is primarily secreted by IFN-γ-activated monocytes, which exert a toxic effect on parasites [46]. In addition, IFN-γ-activated mononuclear cells can enhance phagocytosis for resistance against pathogen invasion. r*Fg*LGMN-1 significantly promoted NO production (40 and 60 μg/mL) and upregulated IFN-γ transcription (all concentrations), and also promoted monocyte phagocytosis (5, 10, 20, 40 μg/mL), which is consistent with the functions of r*Fg*Rab10. Our data indicated that r*Fg*LGMN-1 may have promoted the activation of monocytes, and induced the killing effect of the host against *F. gigantica* invasion. As r*Fg*LGMN-2 inhibited NO production (all concentrations), down-regulated IFN-γ transcription (all concentrations), and inhibited monocyte phagocytosis (10, 40 and 60 μg/mL), it can be speculated that it may inhibit NO secretion and phagocytosis in PBMCs by suppressing IFN-γ transcription, and thereby facilitate the fluke’s survival.

### *Fg*LGMN-1 and *Fg*LGMN-2 interfere with cytokine transcription of PBMCs

Cytokines play critical roles in maintaining host homeostasis during pathogen invasion [47, 48]. In the early stages of *Fasciola* infection, buffaloes exhibit a mixed Th1/Th2 immune response, which may be attributable to the influence of *Fg*ESP [49, 50]. In the present study, r*Fg*LGMN-1 upregulated the transcription of IFN-γ (all concentrations), IL-10 (40 and 60 μg/mL), and TGF-β (10, 20, 40 and 60 μg/mL), while down-regulating IL-4 transcription (5, 40 and 60 μg/mL), which are similar to the effects observed with *Fg*LGMN-1 in Ehsan et al. [51]. These findings suggest that r*Fg*LGMN-1 plays a role in polarizing the immune cytokine profile. IL-4 is essential for mediating humoral immunity and promoting antibody-dependent cell-mediated cytotoxicity [52, 53]; the down-regulation of IL-4 may indicate a reduction in antibody-dependent cell-mediated cytotoxicity efficacy. Furthermore, r*Fg*LGMN-1 likely induces the polarization of PBMCs toward a Th1/Treg immune response. Consequently, r*Fg*LGMN-1 may contribute to the Th1-dominant immune response observed during the early stages of *F. gigantica* infection. Th1 immune responses are known to confer protection against liver fluke infections in livestock [54], and components that drive Th1 polarization may serve as promising vaccine targets [55]. Given the ability of r*Fg*LGMN-1 to induce a Th1 immune response, further exploration of r*Fg*LGMN-1 as a potential vaccine target is warranted.

During the chronic infection stage, *F. gigantica* promotes the secretion of IL-10 and TGF-β through the release of *Fg*ESP, which induces a low-responsive immune state that limits tissue damage and facilitates ongoing infection by flukes [56, 57]. Identifying the components of *Fg*ESP is crucial for the discovery of potentially immunotherapeutic molecules [58]. In the present study, r*Fg*LGMN-2 was shown to inhibit the transcription of IL-4 (10, 20, 40 and 60 μg/mL) and IFN-γ (all concentrations) while promoting the transcription of IL-10 (all concentrations) and TGF-β (10, 20, 40 and 60 μg/mL), similar to the action of *Fg*14-3-3e. These findings suggest that *Fg*LGMN-2 may polarize PBMCs toward a Treg-dominated immune response while inhibiting Th1/Th2 responses. If this is true, r*Fg*LGMN-2 may be one of the components contributing to the low-responsive immune state observed during chronic infection. Given its ability to induce a Treg response, exploring the potential of r*Fg*LGMN-2 as an immunotherapeutic target is a promising avenue for further research.

In this study, we found that r*Fg*LGMN-1 inhibited the proliferation and migration of PBMCs, promoted NO production, and induced Th1 cytokine transcription. r*Fg*LGMN-2 inhibited the proliferation of PBMCs, inhibited NO production, and induced Treg cytokine transcription. These findings suggest diverse roles for r*Fg*LGMN-1 and r*Fg*LGMN-2 in regulating PBMC immune responses. However, given that PBMCs are composed of various cell types, and considering the uncertainties about the dose and timing of natural *Fg*LGMN-1 and *Fg*LGMN-2 secretion in vivo, as well as the differences between recombinant and native *Fg*LGMNs, these results can be challenging to interpret. Therefore, it is necessary to further explore the functions of *Fg*LGMN-1 and *Fg*LGMN-2. Additionally, since 10 *Fg*LGMNs were predicted to be secretory proteins, all members of this protein family need to be investigated to better understand their immunomodulatory roles.

## Conclusions

In the present study, both r*Fg*LGMN-1 and r*Fg*LGMN-2 inhibited the proliferation of PBMCs. While r*Fg*LGMN-1 increased NO production r*Fg*LGMN-2 decreased it. Both r*Fg*LGMN-1 and r*Fg*LGMN-2 increased the transcription of the cytokines IL-10 and TGF-β. However, as there were inherent limitations to the in vitro experiments carried out here such as uncertainties about the dose and timing of natural *Fg*LGMN-1 and *Fg*LGMN-2 secretion in vivo and differences between recombinant and native *Fg*LGMNs. Aadditional research is needed to elucidate the precise functions of *Fg*LGMN-1 and *Fg*LGMN-2.

## Supplementary Information

Additional file 1: Table S1. The primers used in the study

Additional file 2: Table S2. The fragments per kilobase per million reads of 10 *LGMN* transcripts

Additional file 3: Table S3. The differentially transcribed *FgLGMN* transcripts

Additional file 4: Figure S1. Purification and western blot of r*Fg*LGMN-1 and r*Fg*LGMN-2. **a** Purification of r*Fg*LGMN-1.* Lane M* Protein molecular weight standard,* lane 1* flowthrough,* lanes 2–7* imidazole at 8, 10, 20, 100, 200, 500 mM for protein elution. **b** Purification of r*Fg*LGMN-2,* lane 1* flowthrough,* lanes 2–9* imidazole at 8, 10, 20, 40, 60, 80,100, 200, 500 mM for protein elution. **c** Western blot of r*Fg*LGMN-1 electrophoresed under non–reducing conditions and visualized using a chemiluminescent horseradish peroxidase substrate.* Lane M* Protein molecular weight marker.* Lanes 1* and* 2* loaded with r*Fg*LGMN-1,* lane 1* incubation with *Fasciola gigantica*–infected buffalo serum,* lane 2* incubation with *F. gigantica*–negative buffalo serum. **d** Western blot of r*Fg*LGMN-2,* lanes 1* and* 2* loaded with r*Fg*LGMN-2,* lane 1* incubation with *F. gigantica*–infected buffalo serum,* lane 2* incubation with *F. gigantica*–negative buffalo serum

## Data Availability

No datasets were generated or analyzed during the current study.

## Abbreviations

cDNA: Complementary DNA
ConA: Concanavalin A
Ct: Cycle threshold
ESP: Excretory–secretory product
IFN: Interferon
IL: Interleukin
ITS: Internal transcribed spacer
LGMN: Legumain
OD: Optical density
PBMC: Peripheral blood mononuclear cell
PBS: Phosphate-buffered saline
PBST: Phosphate-buffered saline with Tween 20
PCR: Polymerase chain reaction
rDNA: ribosomal DNA
rLGMN: Recombinant legumain
SDS-PAGE: Sodium dodecyl sulfate–polyacrylamide gel electrophoresis
TGF-β: Transforming growth factor β
TPX: Thioredoxin peroxiredoxin
