# Supplementary figures and images for: Immune modulation of buffalo peripheral blood mononuclear cells by two asparaginyl endopeptidases from Fasciola gigantica
Source: Parasit Vectors. 2024 Dec 18;17:516. doi: 10.1186/s13071-024-06570-5 (PMC11656647; doi:10.1186/s13071-024-06570-5)

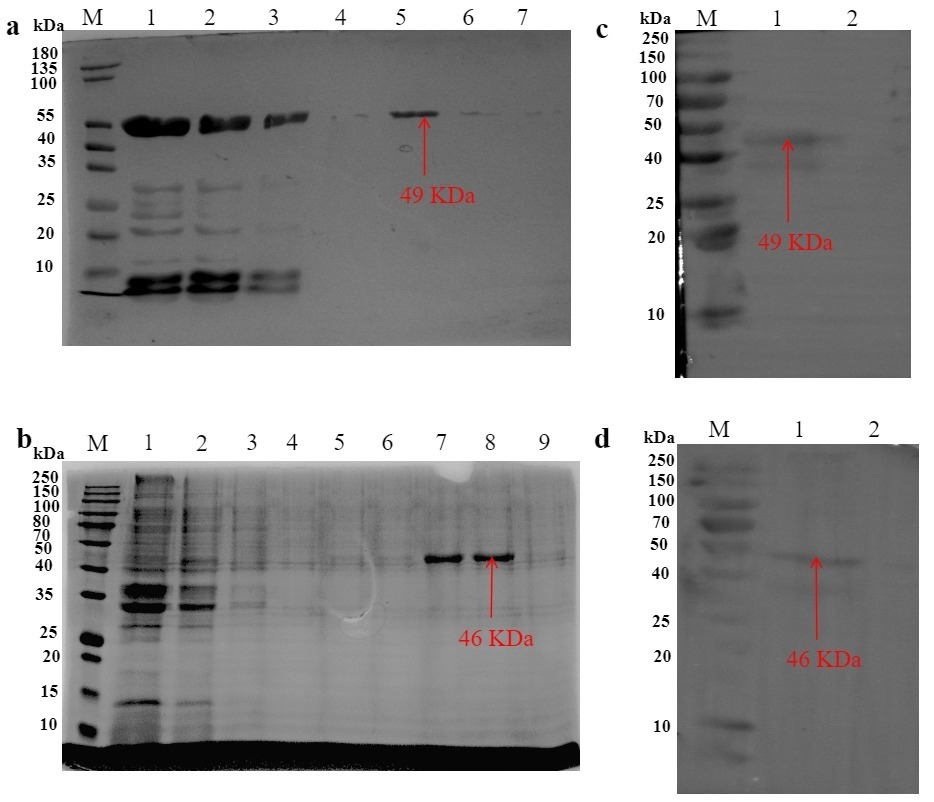

Supplement: Supplementary file 4 — Additional file 4: Figure S1. Purification and western blot of rFgLGMN-1 and rFgLGMN-2. a Purification of rFgLGMN-1. Lane M Protein molecular weight standard, lane 1 flowthrough, lanes 2–7 imidazole at 8, 10, 20, 100, 200, 500 mM for protein elution. b Purification of rFgLGMN-2, lane 1 flowthrough, lanes 2–9 imidazole at 8, 10, 20, 40, 60, 80,100, 200, 500 mM for protein elution. c Western blot of rFgLGMN-1 electrophoresed under non–reducing conditions and visualized using a chemiluminescent horseradish peroxidase substrate. Lane M Protein molecular weight marker. Lanes 1 and 2 loaded with rFgLGMN-1, lane 1 incubation with Fasciola gigantica–infected buffalo serum, lane 2 incubation with F. gigantica–negative buffalo serum. d Western blot of rFgLGMN-2, lanes 1 and 2 loaded with rFgLGMN-2, lane 1 incubation with F. gigantica–infected buffalo serum, lane 2 incubation with F. gigantica–negative buffalo serum [file 13071_2024_6570_MOESM4_ESM.tif]
